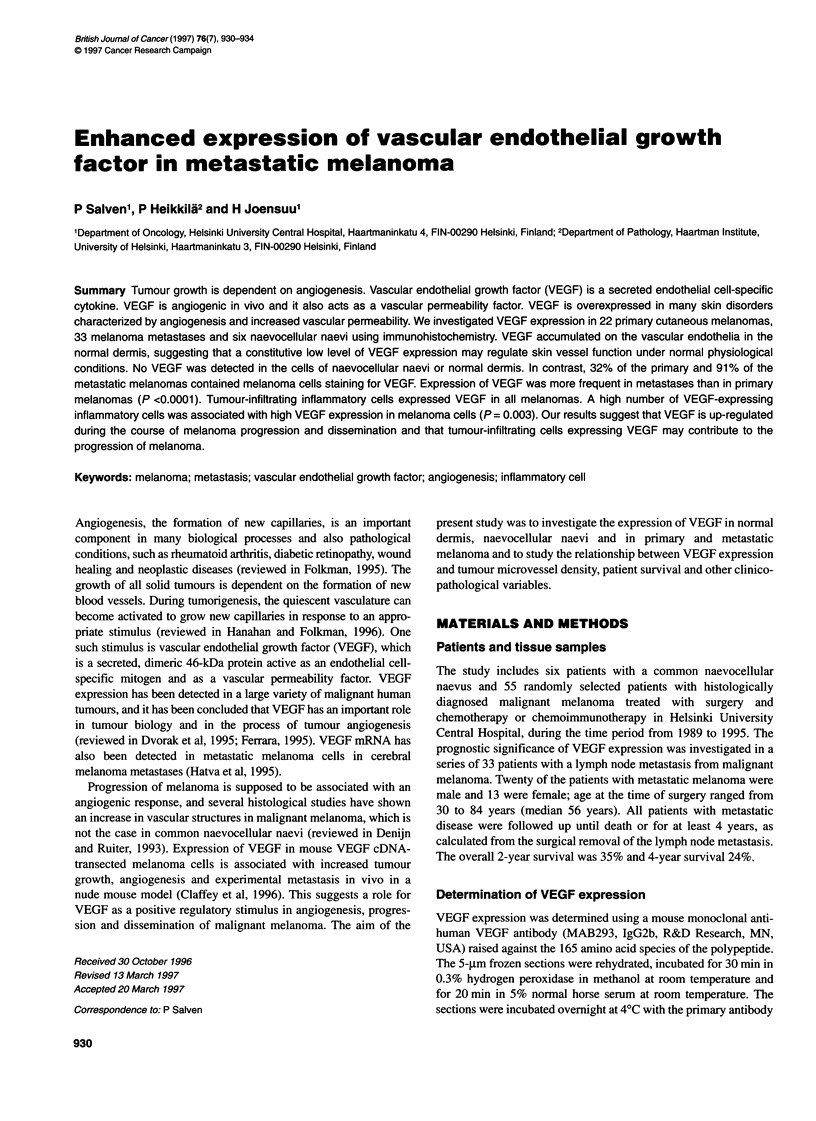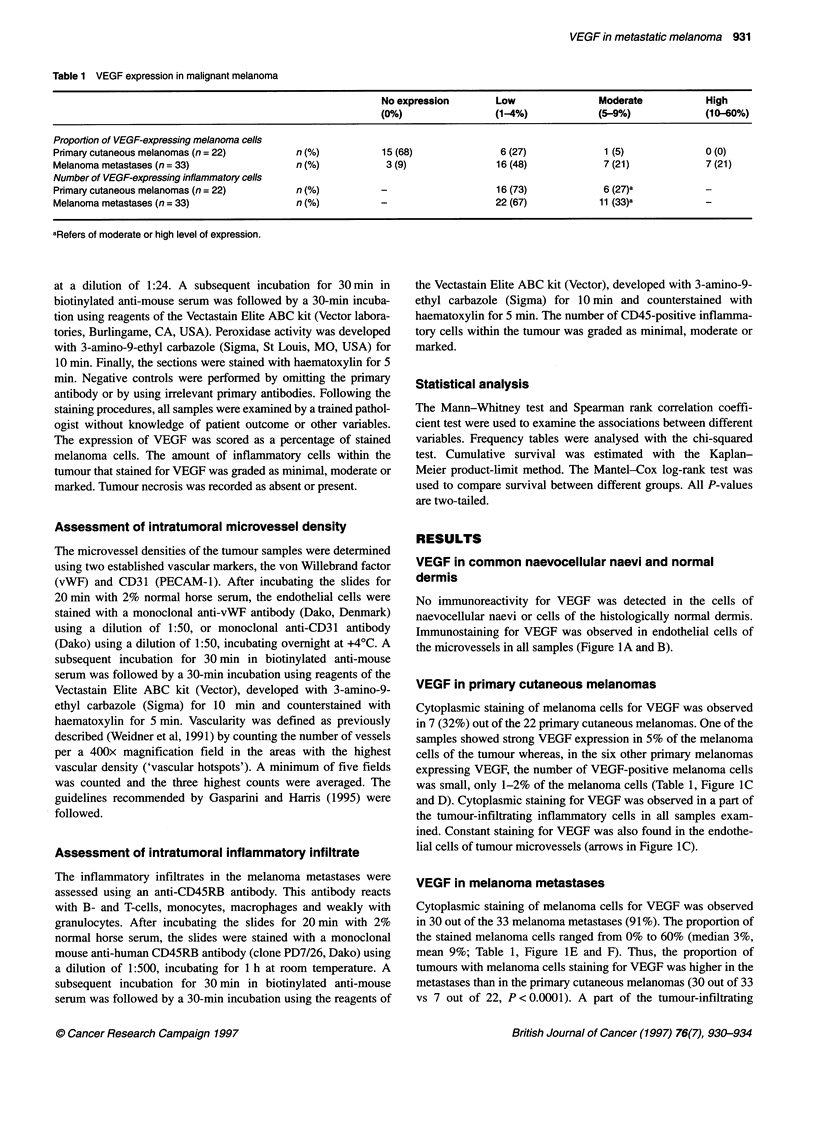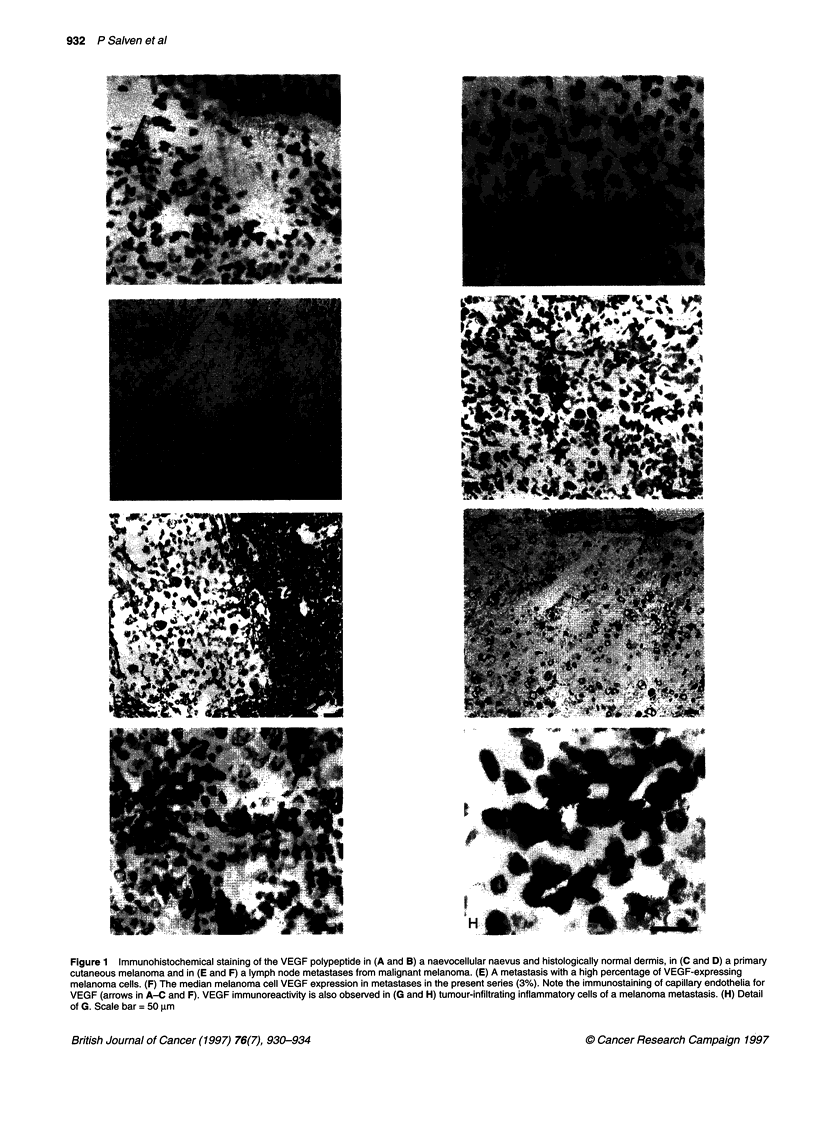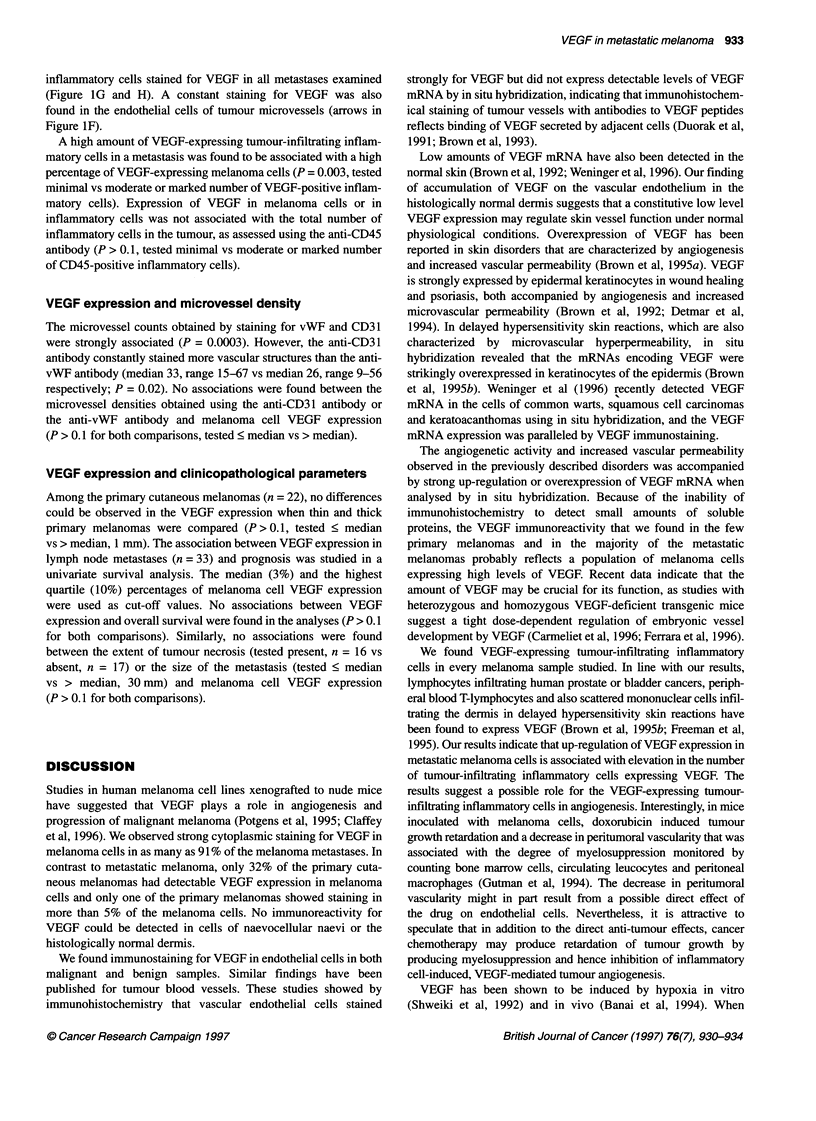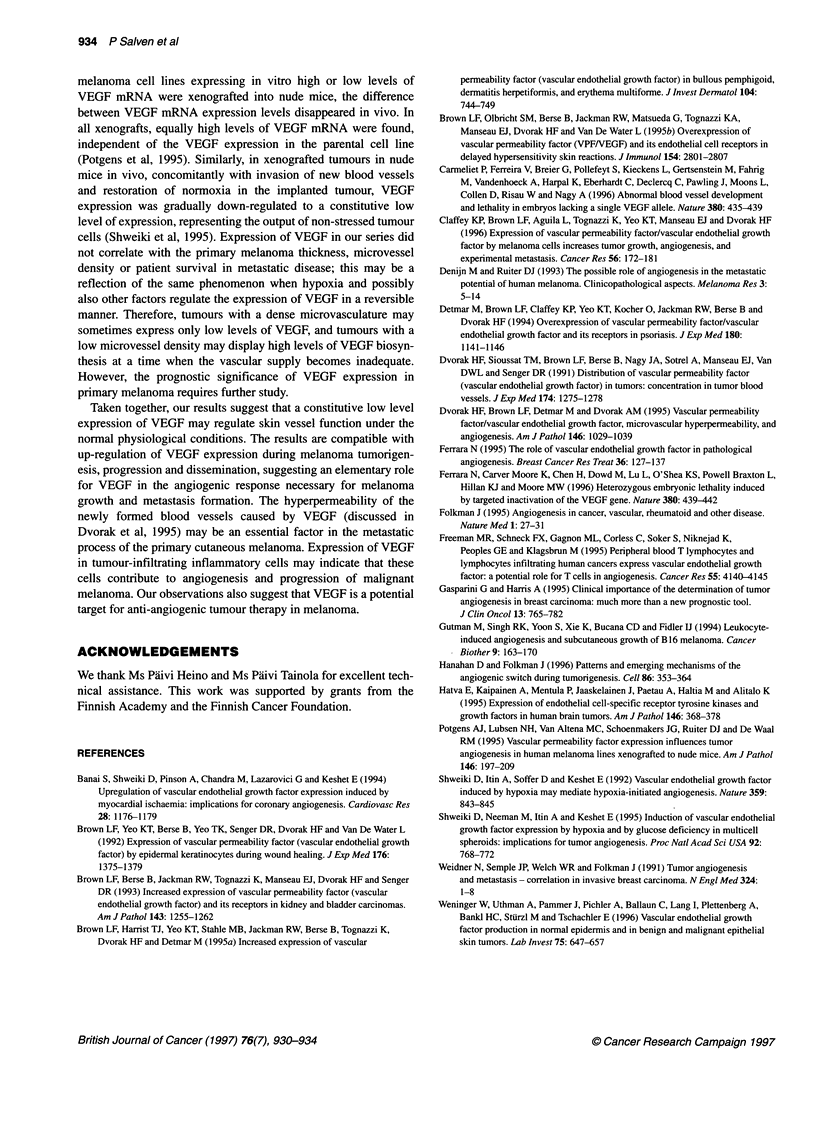# Enhanced expression of vascular endothelial growth factor in metastatic melanoma.

**DOI:** 10.1038/bjc.1997.486

**Published:** 1997

**Authors:** P. Salven, P. HeikkilÃ¤, H. Joensuu

**Affiliations:** Department of Oncology, Helsinki University Central Hospital, Finland.

## Abstract

**Images:**


British Journal of Cancer (1997) 76(7), 930–934
© 1997 Cancer Research Campaign

### Enhanced expression of vascular endothelial growth factor in metastatic melanoma

**P Salven[1], P Heikkilä[2] and H Joensuu[1]**

[1]Department of Oncology, Helsinki University Central Hospital, Haartmaninkatu 4, FIN-00290 Helsinki, Finland; [2]Department of Pathology, Haartman Institute, University of Helsinki, Haartmaninkatu 3, FIN-00290 Helsinki, Finland

**Summary** Tumour growth is dependent on angiogenesis. Vascular endothelial growth factor (VEGF) is a secreted endothelial cell-specific cytokine. VEGF is angiogenic in vivo and it also acts as a vascular permeability factor. VEGF is overexpressed in many skin disorders characterized by angiogenesis and increased vascular permeability. We investigated VEGF expression in 22 primary cutaneous melanomas, 33 melanoma metastases and six naevocellular naevi using immunohistochemistry. VEGF accumulated on the vascular endothelia in the normal dermis, suggesting that a constitutive low level of VEGF expression may regulate skin vessel function under normal physiological conditions. No VEGF was detected in the cells of naevocellular naevi or normal dermis. In contrast, 32% of the primary and 91% of the metastatic melanomas contained melanoma cells staining for VEGF. Expression of VEGF was more frequent in metastases than in primary melanomas ($P < 0.0001$). Tumour-infiltrating inflammatory cells expressed VEGF in all melanomas. A high number of VEGF-expressing inflammatory cells was associated with high VEGF expression in melanoma cells ($P = 0.003$). Our results suggest that VEGF is up-regulated during the course of melanoma progression and dissemination and that tumour-infiltrating cells expressing VEGF may contribute to the progression of melanoma.

**Keywords:** melanoma; metastasis; vascular endothelial growth factor; angiogenesis; inflammatory cell

Angiogenesis, the formation of new capillaries, is an important component in many biological processes and also pathological conditions, such as rheumatoid arthritis, diabetic retinopathy, wound healing and neoplastic diseases (reviewed in Folkman, 1995). The growth of all solid tumours is dependent on the formation of new blood vessels. During tumorigenesis, the quiescent vasculature can become activated to grow new capillaries in response to an appropriate stimulus (reviewed in Hanahan and Folkman, 1996). One such stimulus is vascular endothelial growth factor (VEGF), which is a secreted, dimeric 46-kDa protein active as an endothelial cell-specific mitogen and as a vascular permeability factor. VEGF expression has been detected in a large variety of malignant human tumours, and it has been concluded that VEGF has an important role in tumour biology and in the process of tumour angiogenesis (reviewed in Dvorak et al, 1995; Ferrara, 1995). VEGF mRNA has also been detected in metastatic melanoma cells in cerebral melanoma metastases (Hatva et al, 1995).

Progression of melanoma is supposed to be associated with an angiogenic response, and several histological studies have shown an increase in vascular structures in malignant melanoma, which is not the case in common naevocellular naevi (reviewed in Denijn and Ruiter, 1993). Expression of VEGF in mouse VEGF cDNA-transected melanoma cells is associated with increased tumour growth, angiogenesis and experimental metastasis in vivo in a nude mouse model (Claffey et al, 1996). This suggests a role for VEGF as a positive regulatory stimulus in angiogenesis, progression and dissemination of malignant melanoma. The aim of the present study was to investigate the expression of VEGF in normal dermis, naevocellular naevi and in primary and metastatic melanoma and to study the relationship between VEGF expression and tumour microvessel density, patient survival and other clinicopathological variables.

*Received 30 October 1996*
*Revised 13 March 1997*
*Accepted 20 March 1997*

*Correspondence to:* P Salven

### MATERIALS AND METHODS

### Patients and tissue samples

The study includes six patients with a common naevocellular naevus and 55 randomly selected patients with histologically diagnosed malignant melanoma treated with surgery and chemotherapy or chemoimmunotherapy in Helsinki University Central Hospital, during the time period from 1989 to 1995. The prognostic significance of VEGF expression was investigated in a series of 33 patients with a lymph node metastasis from malignant melanoma. Twenty of the patients with metastatic melanoma were male and 13 were female; age at the time of surgery ranged from 30 to 84 years (median 56 years). All patients with metastatic disease were followed up until death or for at least 4 years, as calculated from the surgical removal of the lymph node metastasis. The overall 2-year survival was 35% and 4-year survival 24%.

### Determination of VEGF expression

VEGF expression was determined using a mouse monoclonal anti-human VEGF antibody (MAB293, IgG2b, R&D Research, MN, USA) raised against the 165 amino acid species of the polypeptide. The 5-µm frozen sections were rehydrated, incubated for 30 min in 0.3% hydrogen peroxidase in methanol at room temperature and for 20 min in 5% normal horse serum at room temperature. The sections were incubated overnight at 4°C with the primary antibody

**Table 1** VEGF expression in malignant melanoma

| | | No expression (0%) | Low (1–4%) | Moderate (5–9%) | High (10–60%) |
|---|---|---|---|---|---|
| *Proportion of VEGF-expressing melanoma cells* | | | | | |
| Primary cutaneous melanomas (*n* = 22) | *n* (%) | 15 (68) | 6 (27) | 1 (5) | 0 (0) |
| Melanoma metastases (*n* = 33) | *n* (%) | 3 (9) | 16 (48) | 7 (21) | 7 (21) |
| *Number of VEGF-expressing inflammatory cells* | | | | | |
| Primary cutaneous melanomas (*n* = 22) | *n* (%) | – | 16 (73) | 6 (27)[a] | – |
| Melanoma metastases (*n* = 33) | *n* (%) | – | 22 (67) | 11 (33)[a] | – |

[a]Refers of moderate or high level of expression.

at a dilution of 1:24. A subsequent incubation for 30 min in biotinylated anti-mouse serum was followed by a 30-min incubation using reagents of the Vectastain Elite ABC kit (Vector laboratories, Burlingame, CA, USA). Peroxidase activity was developed with 3-amino-9-ethyl carbazole (Sigma, St Louis, MO, USA) for 10 min. Finally, the sections were stained with haematoxylin for 5 min. Negative controls were performed by omitting the primary antibody or by using irrelevant primary antibodies. Following the staining procedures, all samples were examined by a trained pathologist without knowledge of patient outcome or other variables. The expression of VEGF was scored as a percentage of stained melanoma cells. The amount of inflammatory cells within the tumour that stained for VEGF was graded as minimal, moderate or marked. Tumour necrosis was recorded as absent or present.

### Assessment of intratumoral microvessel density

The microvessel densities of the tumour samples were determined using two established vascular markers, the von Willebrand factor (vWF) and CD31 (PECAM-1). After incubating the slides for 20 min with 2% normal horse serum, the endothelial cells were stained with a monoclonal anti-vWF antibody (Dako, Denmark) using a dilution of 1:50, or monoclonal anti-CD31 antibody (Dako) using a dilution of 1:50, incubating overnight at +4°C. A subsequent incubation for 30 min in biotinylated anti-mouse serum was followed by a 30-min incubation using reagents of the Vectastain Elite ABC kit (Vector), developed with 3-amino-9-ethyl carbazole (Sigma) for 10 min and counterstained with haematoxylin for 5 min. Vascularity was defined as previously described (Weidner et al, 1991) by counting the number of vessels per a 400× magnification field in the areas with the highest vascular density ('vascular hotspots'). A minimum of five fields was counted and the three highest counts were averaged. The guidelines recommended by Gasparini and Harris (1995) were followed.

### Assessment of intratumoral inflammatory infiltrate

The inflammatory infiltrates in the melanoma metastases were assessed using an anti-CD45RB antibody. This antibody reacts with B- and T-cells, monocytes, macrophages and weakly with granulocytes. After incubating the slides for 20 min with 2% normal horse serum, the slides were stained with a monoclonal mouse anti-human CD45RB antibody (clone PD7/26, Dako) using a dilution of 1:500, incubating for 1 h at room temperature. A subsequent incubation for 30 min in biotinylated anti-mouse serum was followed by a 30-min incubation using the reagents of the Vectastain Elite ABC kit (Vector), developed with 3-amino-9-ethyl carbazole (Sigma) for 10 min and counterstained with haematoxylin for 5 min. The number of CD45-positive inflammatory cells within the tumour was graded as minimal, moderate or marked.

### Statistical analysis

The Mann–Whitney test and Spearman rank correlation coefficient test were used to examine the associations between different variables. Frequency tables were analysed with the chi-squared test. Cumulative survival was estimated with the Kaplan–Meier product-limit method. The Mantel–Cox log-rank test was used to compare survival between different groups. All *P*-values are two-tailed.

### RESULTS

### VEGF in common naevocellular naevi and normal dermis

No immunoreactivity for VEGF was detected in the cells of naevocellular naevi or cells of the histologically normal dermis. Immunostaining for VEGF was observed in endothelial cells of the microvessels in all samples (Figure 1A and B).

### VEGF in primary cutaneous melanomas

Cytoplasmic staining of melanoma cells for VEGF was observed in 7 (32%) out of the 22 primary cutaneous melanomas. One of the samples showed strong VEGF expression in 5% of the melanoma cells of the tumour whereas, in the six other primary melanomas expressing VEGF, the number of VEGF-positive melanoma cells was small, only 1–2% of the melanoma cells (Table 1, Figure 1C and D). Cytoplasmic staining for VEGF was observed in a part of the tumour-infiltrating inflammatory cells in all samples examined. Constant staining for VEGF was also found in the endothelial cells of tumour microvessels (arrows in Figure 1C).

### VEGF in melanoma metastases

Cytoplasmic staining of melanoma cells for VEGF was observed in 30 out of the 33 melanoma metastases (91%). The proportion of the stained melanoma cells ranged from 0% to 60% (median 3%, mean 9%; Table 1, Figure 1E and F). Thus, the proportion of tumours with melanoma cells staining for VEGF was higher in the metastases than in the primary cutaneous melanomas (30 out of 33 vs 7 out of 22, $P < 0.0001$). A part of the tumour-infiltrating

© Cancer Research Campaign 1997

**Figure 1** Immunohistochemical staining of the VEGF polypeptide in (**A** and **B**) a naevocellular naevus and histologically normal dermis, in (**C** and **D**) a primary cutaneous melanoma and in (**E** and **F**) a lymph node metastases from malignant melanoma. (**E**) A metastasis with a high percentage of VEGF-expressing melanoma cells. (**F**) The median melanoma cell VEGF expression in metastases in the present series (3%). Note the immunostaining of capillary endothelia for VEGF (arrows in **A**–**C** and **F**). VEGF immunoreactivity is also observed in (**G** and **H**) tumour-infiltrating inflammatory cells of a melanoma metastasis. (**H**) Detail of **G**. Scale bar = 50 μm

 © Cancer Research Campaign 1997

inflammatory cells stained for VEGF in all metastases examined (Figure 1G and H). A constant staining for VEGF was also found in the endothelial cells of tumour microvessels (arrows in Figure 1F).

A high amount of VEGF-expressing tumour-infiltrating inflammatory cells in a metastasis was found to be associated with a high percentage of VEGF-expressing melanoma cells ($P = 0.003$, tested minimal vs moderate or marked number of VEGF-positive inflammatory cells). Expression of VEGF in melanoma cells or in inflammatory cells was not associated with the total number of inflammatory cells in the tumour, as assessed using the anti-CD45 antibody ($P > 0.1$, tested minimal vs moderate or marked number of CD45-positive inflammatory cells).

### VEGF expression and microvessel density

The microvessel counts obtained by staining for vWF and CD31 were strongly associated ($P = 0.0003$). However, the anti-CD31 antibody constantly stained more vascular structures than the anti-vWF antibody (median 33, range 15–67 vs median 26, range 9–56 respectively; $P = 0.02$). No associations were found between the microvessel densities obtained using the anti-CD31 antibody or the anti-vWF antibody and melanoma cell VEGF expression ($P > 0.1$ for both comparisons, tested ≤ median vs > median).

### VEGF expression and clinicopathological parameters

Among the primary cutaneous melanomas ($n = 22$), no differences could be observed in the VEGF expression when thin and thick primary melanomas were compared ($P > 0.1$, tested ≤ median vs > median, 1 mm). The association between VEGF expression in lymph node metastases ($n = 33$) and prognosis was studied in a univariate survival analysis. The median (3%) and the highest quartile (10%) percentages of melanoma cell VEGF expression were used as cut-off values. No associations between VEGF expression and overall survival were found in the analyses ($P > 0.1$ for both comparisons). Similarly, no associations were found between the extent of tumour necrosis (tested present, $n = 16$ vs absent, $n = 17$) or the size of the metastasis (tested ≤ median vs > median, 30 mm) and melanoma cell VEGF expression ($P > 0.1$ for both comparisons).

### DISCUSSION

Studies in human melanoma cell lines xenografted to nude mice have suggested that VEGF plays a role in angiogenesis and progression of malignant melanoma (Potgens et al, 1995; Claffey et al, 1996). We observed strong cytoplasmic staining for VEGF in melanoma cells in as many as 91% of the melanoma metastases. In contrast to metastatic melanoma, only 32% of the primary cutaneous melanomas had detectable VEGF expression in melanoma cells and only one of the primary melanomas showed staining in more than 5% of the melanoma cells. No immunoreactivity for VEGF could be detected in cells of naevocellular naevi or the histologically normal dermis.

We found immunostaining for VEGF in endothelial cells in both malignant and benign samples. Similar findings have been published for tumour blood vessels. These studies showed by immunohistochemistry that vascular endothelial cells stained strongly for VEGF but did not express detectable levels of VEGF mRNA by in situ hybridization, indicating that immunohistochemical staining of tumour vessels with antibodies to VEGF peptides reflects binding of VEGF secreted by adjacent cells (Duorak et al, 1991; Brown et al, 1993).

Low amounts of VEGF mRNA have also been detected in the normal skin (Brown et al, 1992; Weninger et al, 1996). Our finding of accumulation of VEGF on the vascular endothelium in the histologically normal dermis suggests that a constitutive low level VEGF expression may regulate skin vessel function under normal physiological conditions. Overexpression of VEGF has been reported in skin disorders that are characterized by angiogenesis and increased vascular permeability (Brown et al, 1995*a*). VEGF is strongly expressed by epidermal keratinocytes in wound healing and psoriasis, both accompanied by angiogenesis and increased microvascular permeability (Brown et al, 1992; Detmar et al, 1994). In delayed hypersensitivity skin reactions, which are also characterized by microvascular hyperpermeability, in situ hybridization revealed that the mRNAs encoding VEGF were strikingly overexpressed in keratinocytes of the epidermis (Brown et al, 1995*b*). Weninger et al (1996) recently detected VEGF mRNA in the cells of common warts, squamous cell carcinomas and keratoacanthomas using in situ hybridization, and the VEGF mRNA expression was paralleled by VEGF immunostaining.

The angiogenetic activity and increased vascular permeability observed in the previously described disorders was accompanied by strong up-regulation or overexpression of VEGF mRNA when analysed by in situ hybridization. Because of the inability of immunohistochemistry to detect small amounts of soluble proteins, the VEGF immunoreactivity that we found in the few primary melanomas and in the majority of the metastatic melanomas probably reflects a population of melanoma cells expressing high levels of VEGF. Recent data indicate that the amount of VEGF may be crucial for its function, as studies with heterozygous and homozygous VEGF-deficient transgenic mice suggest a tight dose-dependent regulation of embryonic vessel development by VEGF (Carmeliet et al, 1996; Ferrara et al, 1996).

We found VEGF-expressing tumour-infiltrating inflammatory cells in every melanoma sample studied. In line with our results, lymphocytes infiltrating human prostate or bladder cancers, peripheral blood T-lymphocytes and also scattered mononuclear cells infiltrating the dermis in delayed hypersensitivity skin reactions have been found to express VEGF (Brown et al, 1995*b*; Freeman et al, 1995). Our results indicate that up-regulation of VEGF expression in metastatic melanoma cells is associated with elevation in the number of tumour-infiltrating inflammatory cells expressing VEGF. The results suggest a possible role for the VEGF-expressing tumour-infiltrating inflammatory cells in angiogenesis. Interestingly, in mice inoculated with melanoma cells, doxorubicin induced tumour growth retardation and a decrease in peritumoral vascularity that was associated with the degree of myelosuppression monitored by counting bone marrow cells, circulating leucocytes and peritoneal macrophages (Gutman et al, 1994). The decrease in peritumoral vascularity might in part result from a possible direct effect of the drug on endothelial cells. Nevertheless, it is attractive to speculate that in addition to the direct anti-tumour effects, cancer chemotherapy may produce retardation of tumour growth by producing myelosuppression and hence inhibition of inflammatory cell-induced, VEGF-mediated tumour angiogenesis.

VEGF has been shown to be induced by hypoxia in vitro (Shweiki et al, 1992) and in vivo (Banai et al, 1994). When

© Cancer Research Campaign 1997

melanoma cell lines expressing in vitro high or low levels of VEGF mRNA were xenografted into nude mice, the difference between VEGF mRNA expression levels disappeared in vivo. In all xenografts, equally high levels of VEGF mRNA were found, independent of the VEGF expression in the parental cell line (Potgens et al, 1995). Similarly, in xenografted tumours in nude mice in vivo, concomitantly with invasion of new blood vessels and restoration of normoxia in the implanted tumour, VEGF expression was gradually down-regulated to a constitutive low level of expression, representing the output of non-stressed tumour cells (Shweiki et al, 1995). Expression of VEGF in our series did not correlate with the primary melanoma thickness, microvessel density or patient survival in metastatic disease; this may be a reflection of the same phenomenon when hypoxia and possibly also other factors regulate the expression of VEGF in a reversible manner. Therefore, tumours with a dense microvasculature may sometimes express only low levels of VEGF, and tumours with a low microvessel density may display high levels of VEGF biosynthesis at a time when the vascular supply becomes inadequate. However, the prognostic significance of VEGF expression in primary melanoma requires further study.

Taken together, our results suggest that a constitutive low level expression of VEGF may regulate skin vessel function under the normal physiological conditions. The results are compatible with up-regulation of VEGF expression during melanoma tumorigenesis, progression and dissemination, suggesting an elementary role for VEGF in the angiogenic response necessary for melanoma growth and metastasis formation. The hyperpermeability of the newly formed blood vessels caused by VEGF (discussed in Dvorak et al, 1995) may be an essential factor in the metastatic process of the primary cutaneous melanoma. Expression of VEGF in tumour-infiltrating inflammatory cells may indicate that these cells contribute to angiogenesis and progression of malignant melanoma. Our observations also suggest that VEGF is a potential target for anti-angiogenic tumour therapy in melanoma.

### ACKNOWLEDGEMENTS

We thank Ms Päivi Heino and Ms Päivi Tainola for excellent technical assistance. This work was supported by grants from the Finnish Academy and the Finnish Cancer Foundation.

### REFERENCES

Banai S, Shweiki D, Pinson A, Chandra M, Lazarovici G and Keshet E (1994) Upregulation of vascular endothelial growth factor expression induced by myocardial ischaemia: implications for coronary angiogenesis. *Cardiovasc Res* **28**: 1176–1179

Brown LF, Yeo KT, Berse B, Yeo TK, Senger DR, Dvorak HF and Van De Water L (1992) Expression of vascular permeability factor (vascular endothelial growth factor) by epidermal keratinocytes during wound healing. *J Exp Med* **176**: 1375–1379

Brown LF, Berse B, Jackman RW, Tognazzi K, Manseau EJ, Dvorak HF and Senger DR (1993) Increased expression of vascular permeability factor (vascular endothelial growth factor) and its receptors in kidney and bladder carcinomas. *Am J Pathol* **143**: 1255–1262

Brown LF, Harrist TJ, Yeo KT, Stahle MB, Jackman RW, Berse B, Tognazzi K, Dvorak HF and Detmar M (1995*a*) Increased expression of vascular permeability factor (vascular endothelial growth factor) in bullous pemphigoid, dermatitis herpetiformis, and erythema multiforme. *J Invest Dermatol* **104**: 744–749

Brown LF, Olbricht SM, Berse B, Jackman RW, Matsueda G, Tognazzi KA, Manseau EJ, Dvorak HF and Van De Water L (1995*b*) Overexpression of vascular permeability factor (VPF/VEGF) and its endothelial cell receptors in delayed hypersensitivity skin reactions. *J Immunol* **154**: 2801–2807

Carmeliet P, Ferreira V, Breier G, Pollefeyt S, Kieckens L, Gertsenstein M, Fahrig M, Vandenhoeck A, Harpal K, Eberhardt C, Declercq C, Pawling J, Moons L, Collen D, Risau W and Nagy A (1996) Abnormal blood vessel development and lethality in embryos lacking a single VEGF allele. *Nature* **380**: 435–439

Claffey KP, Brown LF, Aguila L, Tognazzi K, Yeo KT, Manseau EJ and Dvorak HF (1996) Expression of vascular permeability factor/vascular endothelial growth factor by melanoma cells increases tumor growth, angiogenesis, and experimental metastasis. *Cancer Res* **56**: 172–181

Denijn M and Ruiter DJ (1993) The possible role of angiogenesis in the metastatic potential of human melanoma. Clinicopathological aspects. *Melanoma Res* **3**: 5–14

Detmar M, Brown LF, Claffey KP, Yeo KT, Kocher O, Jackman RW, Berse B and Dvorak HF (1994) Overexpression of vascular permeability factor/vascular endothelial growth factor and its receptors in psoriasis. *J Exp Med* **180**: 1141–1146

Dvorak HF, Sioussat TM, Brown LF, Berse B, Nagy JA, Sotrel A, Manseau EJ, Van DWL and Senger DR (1991) Distribution of vascular permeability factor (vascular endothelial growth factor) in tumors: concentration in tumor blood vessels. *J Exp Med* **174**: 1275–1278

Dvorak HF, Brown LF, Detmar M and Dvorak AM (1995) Vascular permeability factor/vascular endothelial growth factor, microvascular hyperpermeability, and angiogenesis. *Am J Pathol* **146**: 1029–1039

Ferrara N (1995) The role of vascular endothelial growth factor in pathological angiogenesis. *Breast Cancer Res Treat* **36**: 127–137

Ferrara N, Carver Moore K, Chen H, Dowd M, Lu L, O'Shea KS, Powell Braxton L, Hillan KJ and Moore MW (1996) Heterozygous embryonic lethality induced by targeted inactivation of the VEGF gene. *Nature* **380**: 439–442

Folkman J (1995) Angiogenesis in cancer, vascular, rheumatoid and other disease. *Nature Med* **1**: 27–31

Freeman MR, Schneck FX, Gagnon ML, Corless C, Soker S, Niknejad K, Peoples GE and Klagsbrun M (1995) Peripheral blood T lymphocytes and lymphocytes infiltrating human cancers express vascular endothelial growth factor: a potential role for T cells in angiogenesis. *Cancer Res* **55**: 4140–4145

Gasparini G and Harris A (1995) Clinical importance of the determination of tumor angiogenesis in breast carcinoma: much more than a new prognostic tool. *J Clin Oncol* **13**: 765–782

Gutman M, Singh RK, Yoon S, Xie K, Bucana CD and Fidler IJ (1994) Leukocyte-induced angiogenesis and subcutaneous growth of B16 melanoma. *Cancer Biother* **9**: 163–170

Hanahan D and Folkman J (1996) Patterns and emerging mechanisms of the angiogenic switch during tumorigenesis. *Cell* **86**: 353–364

Hatva E, Kaipainen A, Mentula P, Jaaskelainen J, Paetau A, Haltia M and Alitalo K (1995) Expression of endothelial cell-specific receptor tyrosine kinases and growth factors in human brain tumors. *Am J Pathol* **146**: 368–378

Potgens AJ, Lubsen NH, Van Altena MC, Schoenmakers JG, Ruiter DJ and De Waal RM (1995) Vascular permeability factor expression influences tumor angiogenesis in human melanoma lines xenografted to nude mice. *Am J Pathol* **146**: 197–209

Shweiki D, Itin A, Soffer D and Keshet E (1992) Vascular endothelial growth factor induced by hypoxia may mediate hypoxia-initiated angiogenesis. *Nature* **359**: 843–845

Shweiki D, Neeman M, Itin A and Keshet E (1995) Induction of vascular endothelial growth factor expression by hypoxia and by glucose deficiency in multicell spheroids: implications for tumor angiogenesis. *Proc Natl Acad Sci USA* **92**: 768–772

Weidner N, Semple JP, Welch WR and Folkman J (1991) Tumor angiogenesis and metastasis – correlation in invasive breast carcinoma. *N Engl Med* **324**: 1–8

Weninger W, Uthman A, Pammer J, Pichler A, Ballaun C, Lang I, Plettenberg A, Bankl HC, Stürzl M and Tschachler E (1996) Vascular endothelial growth factor production in normal epidermis and in benign and malignant epithelial skin tumors. *Lab Invest* **75**: 647–657

© Cancer Research Campaign 1997